# The Wnt Receptor, Lrp5, Is Expressed by Mouse Mammary Stem Cells and Is Required to Maintain the Basal Lineage

**DOI:** 10.1371/journal.pone.0006594

**Published:** 2009-08-12

**Authors:** Nisha M. Badders, Shruti Goel, Rod J. Clark, Kristine S. Klos, Soyoung Kim, Anna Bafico, Charlotta Lindvall, Bart O. Williams, Caroline M. Alexander

**Affiliations:** 1 McArdle Laboratory for Cancer Research, University of Wisconsin-Madison, Madison, Wisconsin, United States of America; 2 Department of Pathology, Cardiff University, Cardiff, Wales, United Kingdom; 3 Laboratory of Cell Signaling and Carcinogenesis, Van Andel Research Institute, Grand Rapids, Michigan, United States of America; City of Hope Medical Center, United States of America

## Abstract

**Background:**

Ectopic Wnt signaling induces increased stem/progenitor cell activity in the mouse mammary gland, followed by tumor development. The Wnt signaling receptors, Lrp5/6, are uniquely required for canonical Wnt activity. Previous data has shown that the absence of *Lrp5* confers resistance to Wnt1-induced tumor development.

**Methodology/Principal Findings:**

Here, we show that all basal mammary cells express Lrp5, and co-express Lrp6 in a similar fashion. Though Wnt dependent transcription of key target genes is relatively unchanged in mammary epithelial cell cultures, the absence of Lrp5 specifically depletes adult regenerative stem cell activity (to less than 1%). Stem cell activity can be enriched by >200 fold (over 80% of activity), based on high Lrp5 expression alone. Though *Lrp5* null glands have apparent normal function, the basal lineage is relatively reduced (from 42% basal/total epithelial cells to 22%) and Lrp5−/− mammary epithelial cells show enhanced expression of senescence-associated markers *in vitro*, as measured by expression of p16^Ink4a^ and TA-p63.

**Conclusions/Significance:**

This is the first single biomarker that has been demonstrated to be functionally involved in stem cell maintenance. Together, these results demonstrate that Wnt signaling through Lrp5 is an important component of normal mammary stem cell function.

## Introduction

The Wnt signaling pathway is required for normal development, but when ectopically expressed, is highly oncogenic for human epithelia [1]. Wnt signaling is used at many different developmental stages, as an effector of pathways involved in processes as distinct as planar cell polarity, neuronal axon guidance and the activation and regulation of somatic epithelial stem/progenitor cell compartments [2], [3], [4], [5]. This latter function appears to be key to the oncogenic role of Wnt signaling. In gut, the mutation of key tumor suppressor molecules in the Wnt signaling pathway leads to amplification of stem/progenitor compartments, followed by the appearance of differentiated adenomas and tumors [6], [7], [8]. Our previous data has shown that gain of function of Wnt signaling in mammary glands also induces an increase in the stem/progenitor cell activity in the preneoplastic condition [9].

In order to understand the normal function of Wnt signaling in mammary glands, we chose to study how loss of function of Wnt signaling affected mammary development.

There are many Wnt-dependent signaling events, but only one pathway has so far been associated with the stem cell functions and oncogenic properties. This so-called canonical pathway is mediated by the interaction of Wnt ligands with a pair of cell surface receptors, comprising a Frizzled receptor and an Lrp5 or -6 receptor [10], [11]. Binding of the Wnt ligand to the Frizzled and Lrp5/6 receptor is followed by the recruitment of axin from the β-catenin destruction complex, stabilization of β-catenin and, transactivation of specific target genes via a β-catenin/TCF complex [12].

There are many members of the Wnt family of secreted lipoglycoprotein ligands [13], and several (Wnt-2,-4,-5a,-5b,6,7b) are expressed during mammary gland development [14], [15], [16], [17], [18]. Similarly, there are 10 known Frizzled homologues, of which Frizzled 1–8 are known to be expressed in mammary epithelial cells [19]. However, there is an absolute requirement for either Lrp5 or Lrp6 for canonical Wnt signaling [20]. Lrp5 and -6 belong to the LDL receptor related protein family of single-span transmembrane receptors, which mediate binding and internalization of various lipoprotein particles [21].

Current studies have not addressed whether Lrp5 and Lrp6 have distinct molecular properties. Ablation of *Lrp5* and *Lrp6* produce entirely different phenotypes in mice. Lrp6 expression appears to be widespread in embryonic tissues and is essential for embryonic development. Mammary development fails in the absence of Lrp6; both epithelial outgrowth of the placode and the formation of the host adipose tissue is affected [22]. The role of Lrp6 in adult tissues is unclear, but loss of function mutations have been linked with human cases of coronary artery disease [23]. In contrast, *Lrp5* null mice are viable, although they exhibit defects in bone ossification and vascularization of the eye [24], [25]. In adult tissues, Lrp5 mRNA and protein levels are high and widely expressed in tissues such as bone, pancreas, central nervous system, and in phagocytic cells [21], [26]. Loss of function mutations have been associated with heritable cases of osteoporosis as well as Type I diabetes [27], [28].

In the mammary gland, Wnt signaling is required for specification and outgrowth of the mammary rudiment from the embryonic skin [16], and a Wnt reporter strain shows high Wnt signaling activity at this stage [15], [29]. Since inhibition of Wnt signaling prevents gland formation [15], it has been difficult to determine the functional role of Wnt signaling in later and adult stages of mammary gland development.

Wnt signaling has been shown to be important not only to the maintenance of stem/progenitor compartments in gut, but in a number of other cell lineages. These include hematopoetic and embryonic stem cells [30], [31], [32]. Specifically, several components of the canonical Wnt signaling pathway have been found to be expressed in both embryonic and hematopoetic stem cell populations. Moreover, treatment with Wnt ligands or downstream activation of the Wnt signaling pathway inhibits differentiation and promotes self-renewal of these cells [30], [31]. Studies published in 2006 [33], [34] showed that subpopulations of basal mammary cells could be isolated from the total population, that show enhanced regenerative capacity when assayed *in vivo* (described by their ability to regenerate a mammary tree when transferred to a host cleared fat pad). A single cell from this population was sufficient to recreate a whole gland, and they were coined somatic mammary stem cells. These subpopulations are separated by their high expression of both CD24 and CD49f (α6 integrin, or CD29, β1 integrin), but their purity is unlikely to be higher than 5%. Neither of these markers alone is useful for the identification of stem cells, or indeed resolution of whole mammary epithelial cell populations. Therefore, the behavior of the cells that are key to the growth or regeneration of glands has not yet been described. It has become a high priority to find a molecule (preferably one functionally involved in determining stemness) that is a specific marker of stem cell function, for their evaluation during normal and pathogenic development.

Previously, we showed that *Lrp5* null mammary glands, though grossly normal (albeit developmentally delayed), were remarkably resistant to Wnt1-induced tumor development [29]. This resistance occurred despite the presence of Lrp6, and served to focus our attention on the specific functions of Lrp5. *Lrp5* null glands were almost devoid of regenerative potential when tested by *in vivo* stem cell assay. Here, we show that both Lrp5 and -6 proteins are expressed in the basal epithelial cell population. We also show that the loss of *Lrp5* does not significantly affect the response of cultured mammary epithelial cells (MECs), tested with an *in vitro* Wnt reporter assay. The absence of *Lrp5* generates a selective loss of the basal cell population, though the function of mammary glands is entirely preserved. Furthermore, the cells tend to become senescent in culture. In addition, we find that cells expressing high levels of Lrp5 co-localize with the CD24/CD49f double-positive stem cell-enriched fraction and have enhanced stem cell function *in vivo*.

## Results

### Lrp5 and 6 Expression is Localized to Basal Mammary Epithelial Cells

Lrp5 and Lrp6 proteins contain >70% amino acid sequence homology [21]. Prior studies have shown no clear patterns of expression of Lrp5 and -6, depending upon the antibody reagents used, the assay, and the fixation conditions. To ensure that our analyses can resolve Lrp5 and Lrp6, we transfected HEK293 cells with constructs coding for either Lrp5 or Lrp6. Western blotting, immunofluorescence, and flow cytometry staining of transfected 293 cells found both Lrp5 and Lrp6 antibodies were specific to each receptor (Figure S1).

**Figure 1:**
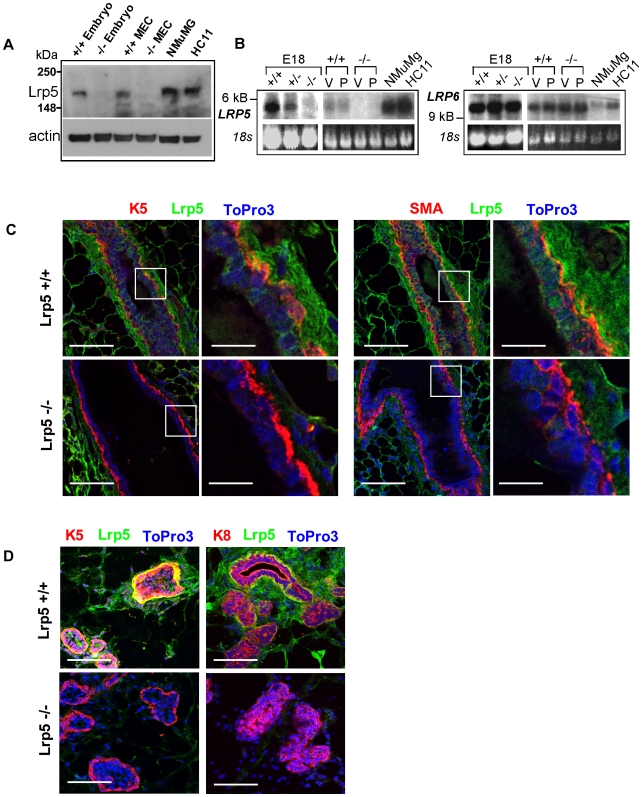
Lrp5 is Expressed in Mouse Mammary Epithelial Cells. A) Western blot of lysates prepared from E10-12 *Lrp5* +/+ and −/− embryos, primary MECs, and the NMuMG and HC11 cell lines. B) Northern blots of RNA prepared from E18 *Lrp5* +/+, +/−, −/− embryos, primary virgin (V) and 14 day pregnant (P) MECs, NMuMG and HC11 cell lines were probed for either Lrp5 or Lrp6 mRNA expression. 18S was used as a loading control. C) Immunohistochemistry was used to localize Lrp5-expressing cells in virgin *Lrp5* +/+ and −/− paraffin embedded mammary glands. Lrp5 (green) was co-localized with K5 or SMA (red), and nuclei stained with ToPro3 (blue) as indicated, scale bars = 20 µm. Insets depict 5×enlargements, scale bars = 4 µm. D) Frozen sections of 10 day pregnant mammary glands from *Lrp5* +/+ and −/− mice were subjected to the same analysis, scale bars = 20 µm.

By immunofluorescent assay, quiescent virgin glands showed specific staining of Lrp5 in basal epithelial cells (determined by their co-expression of either keratin 5 (K5) or smooth muscle actin (SMA); Fig. 1), but not luminal cells. Interestingly, we did observe specific staining in the stroma of Lrp5 +/+ mammary glands, which was absent in Lrp5 −/− glands. There was high background staining of the adipocytes in these paraffin-embedded samples, demonstrated by a persistent staining pattern in these cells in *Lrp5*−/− glands, where the specific basal cell-associated stain is absent. Overall, ductal development and morphogenesis in Lrp5−/− mammary glands is almost normal (a little delayed and hypo-branched; Lindvall et al 2006). These immunohistochemical data show that the relative cellular architecture and arrangement is also normal.

In frozen sections of mammary glands from pregnant mice, we confirmed that the morphogenesis and growth typical of this stage is normal, and that the basal cell pattern of expression was continued (co-localization with basal markers, and exclusion from luminal, keratin 8 (K8)-positive cells).

Northern analysis of mRNA and Western analysis of proteins from embryos and isolated MECs confirmed the loss of *Lrp5* expression in these mice (Fig. 1B). Lrp5 mRNA was expressed at similar levels in glands from both virgin and pregnant mice. In the absence of Lrp5, there was no significant compensatory upregulation of Lrp6 (at the mRNA level)

**Figure 2:**
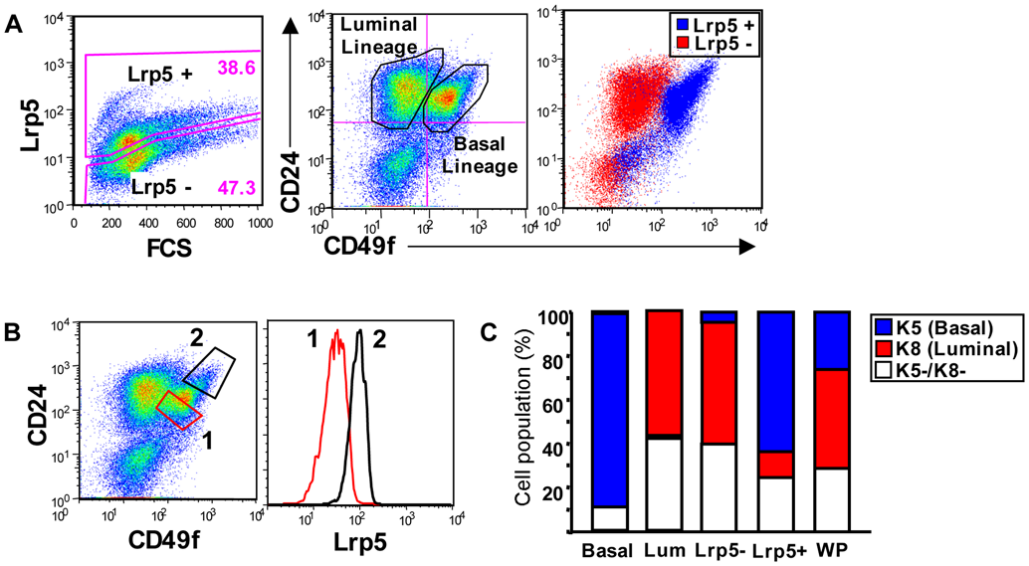
Lrp5 is Expressed by Basal Mammary Epithelial Cells. A) *BALB/c* virgin MECs were stained with CD24, CD49f, and Lrp5 prior to FACS analysis. MECs were gated for positive (blue) and negative (red) Lrp5 expression and the subsequent CD24/CD49f profile of the populations were overlaid. B) Basal MECs were gated based on the magnitude of CD24/CD49f expression level, as CD24^low^/CD49f^low^ (1, red) or CD24^high^/CD49f^high^ (2, black). The relative Lrp5 expression levels of each population were then overlaid as histograms. C) Basal, Luminal, Lrp5+, Lrp5-, and whole population (WP) MEC fractions (gates show in A) were isolated by FACS and subsequently stained for K5 and K8 to confirm their purity and cellular phenotype.

To verify that Lrp5 was expressed in basal cells, we used the flow cytometric analysis of *BALB/c* MECs described by Stingl et al, separating basal and luminal cells by their dual staining with CD24 and CD49f [34]. Analysis of Lrp5 expression in addition to CD24 and CD49f revealed that Lrp5 expression was predominantly localized to the basal lineage (Fig. 2A). Though the intensity of Lrp5 staining was much lower in *C57Bl6* MECs, analysis of this strain also showed that only basal cells expressed Lrp5, and that this staining was absent in the corresponding *C57Bl6 Lrp5* −/− MECs (Figure S2).

The expression of Lrp5 was not equal in all basal cells; indeed the non-regenerative myoepithelial population (relatively lower CD24/CD49f; Stingl et al 2006) had low Lrp5 expression, whereas the regenerative mammary repopulating units (MRU) fraction had higher Lrp5 expression levels (Fig. 2A and B). Isolation of cell fractions by flow cytometry, followed by staining of sorted populations with antibodies to keratin 5 (K5) and -8 (K8) confirmed that the accuracy of gating for the basal and luminal cell populations was high. Likewise, isolation and staining of the Lrp5-positive and -negative populations for K5 and K8 showed that the Lrp5-positive population consists predominantly of basal cells and the Lrp5 negative population contains mostly luminal cells (Fig. 2C).

**Figure 3:**
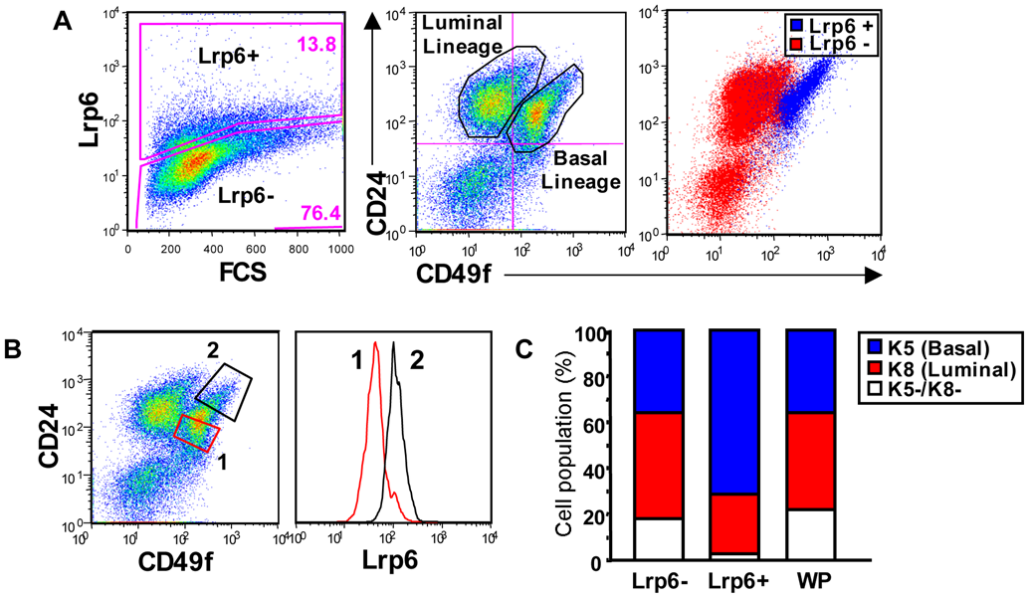
Lrp6 is co-expressed by Basal Mammary Epithelial Cells. A) *BALB/c* virgin MECs were stained with CD24, CD49f, and Lrp6 prior to FACS analysis. MECs were divided into Lrp6+/high and Lrp6-/low, and these populations were overlaid onto the CD24/CD49f profile (Lrp6+ in blue, Lrp6- in red). B) Basal MECs were analyzed as described in Fig. 2b, to test the magnitude of Lrp6 expression relative to CD24/CD49f. C) Lrp6+, Lrp6-, and whole population (WP) MEC fractions were isolated by FACS and subsequently stained for K5 and K8 to determine their cellular phenotype.

Staining of *BALB/c* MECs with anti-Lrp6, in addition to CD24 and CD49f, demonstrated that Lrp6 is also predominantly localized to the basal cell lineage (Fig. 3A) and the magnitude of Lrp6 expression is greater in basal cells expressing high levels of CD49f and CD24 (Fig. 3B). Furthermore, staining of isolated Lrp6 positive cells for K5 and K8 showed that these cells are predominantly basal (Fig. 3C). We conclude that most basal cells express both Lrp5 and Lrp6.

### High Lrp5 Expression Identifies a Stem Cell Enriched Population

**Figure 4:**
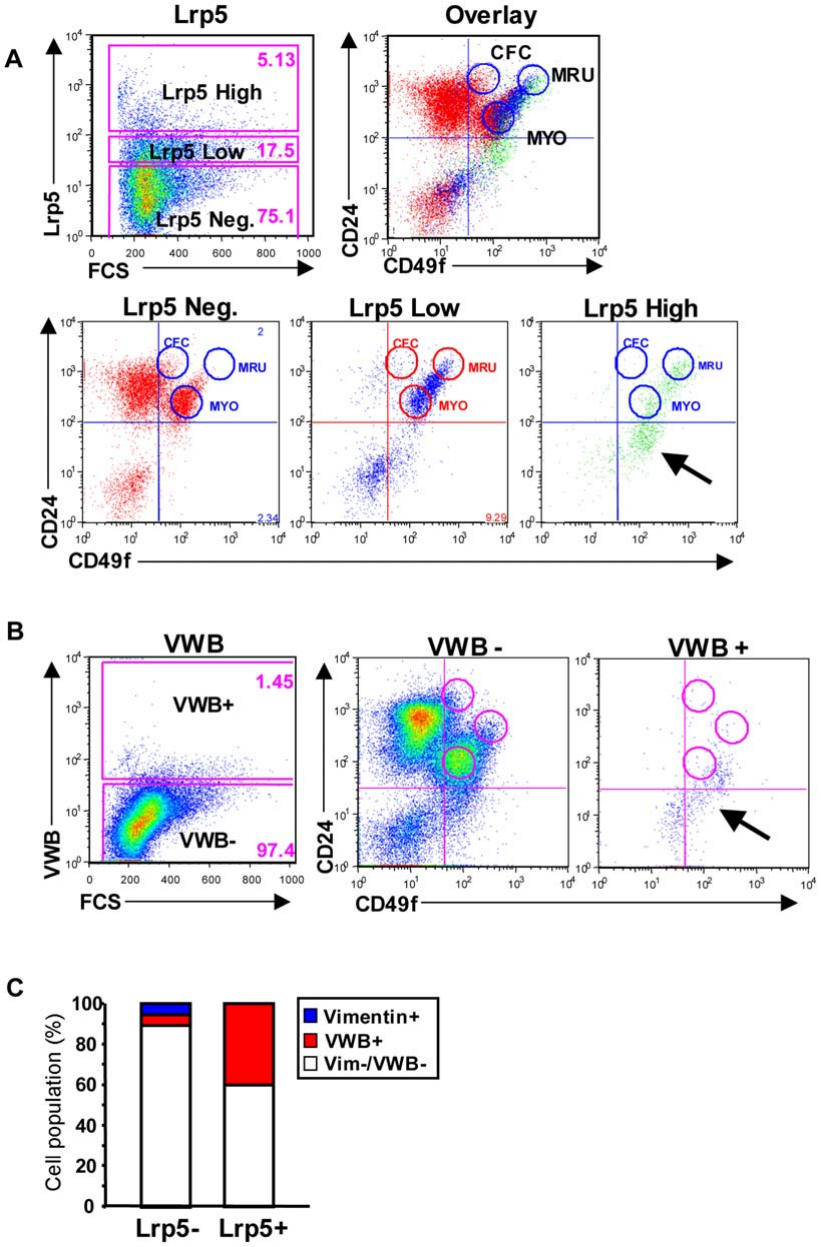
Lrp5 is Expressed by Cells that Co-Purify with Stem Cells by Flow Cytometry. A) Single cell preparations of virgin *BALB/c* MECs were stained with CD24, CD49f, CD45, and CD31, in addition to Lrp5 prior to FACS analysis. Gates were set to distinguish Lrp5 negative (red), low (blue), and high (green) expressing MECs. The CD24/CD49f profiles for each population were then overlaid. Arrow in Lrp5 high panel indicates previously uncharacterized region of CD24/CD49f profile. B) MECs were stained with von Willebrand factor (VWB), in addition to CD49f and CD24. Gates were drawn to indicate VWB positive and negative populations and the CD24/CD49f profiles were plotted for each respective population. Arrow indicates VWB+ cell population that co-localizes with a fraction of Lrp5 high MECs shown in A. C) Lrp5+ and Lrp5- MEC fractions were isolated by FACS and stained for Vimentin and VWB expression.

Previously, CD24^+^CD49f^high^ antibody staining has been shown to identify a population of MECs highly enriched in stem cell activity, termed mammary repopulating units (MRU) [33], [34]. The stem cell frequency of the MRU fraction was estimated to be 1/60–1/90 cells based on ductal outgrowth upon transplantation into cleared fat pads [34], [35]. To evaluate the functional stem cell activity of Lrp5-expressing cells, we separated high- and low Lrp5-expressing cells. Flow cytometric analysis using differential C49f/CD24 staining pattern showed that the Lrp5-high cells were enriched in the MRU fraction (Fig. 4A). Approximately 50% of Lrp5-high cells were distributed to a previously undefined region of the CD24/CD49f FACS profile. Further analysis revealed this fraction primarily contained von Willebrand factor (VWB) positive endothelial cells (Fig. 4B). Although CD31 positive cells were excluded by previous gating, sorting and staining of Lrp5 negative and positive populations, confirmed that approximately 40% of the Lrp5-positive cell fraction was VWB positive (Fig. 4C).

**Figure 5:**
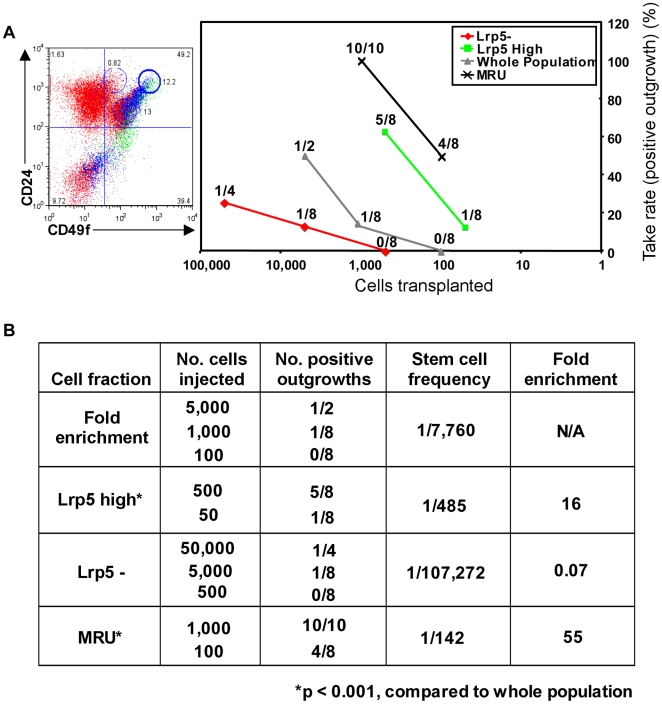
Lrp5-High Cells are Enriched for Stem Cell Activity. A) Mammary epithelial cells were isolated from 10-week, virgin *BALB/c* mice, stained for Lrp5 and FACS sorted, as described above. Lrp5 high (red) and negative (green) MECs were then transplanted into cleared fat pads of 3-week *BALB/c* recipient mice. Following 8 weeks, mammary glands were harvested, carmine stained, and scored for primary outgrowths, as described above. Transplantations of the MRU sub-population (black), in addition to the FACS sorted, unfractionated population (Whole Population, gray) served as controls. Left panel is a representative CD24/CD49f FACS profile, depicting overlaid Lrp5- (red), Lrp5 low (blue), and Lrp5 high (green) populations. B) Estimation of stem cell frequencies for each transplanted population. Stem cell frequencies, fold enrichment, and p values were calculated using limdil software (http://bioinf.wehi.edu.au/software/limdil).

Transplantation of Lrp5 high cells in limiting dilutions into cleared fat pads demonstrated that Lrp5 high cells possess significantly augmented ductal stem cell activity compared to the whole population (Fig. 5A). The estimated stem cell frequency was found to increase from 1/7,760 cells in the whole population to 1/485 in the Lrp5-high fraction (a 16-fold enrichment in mammary stem cells; Fig. 5B). In addition, Lrp5 negative cells were depleted of ductal stem cell activity (1/107,272 cells) compared to the whole population, and the overall recovery of stem cell function in the Lrp5-high fraction was 80%. Based on this one marker (and assuming a 50% dilution with VWB-positive endothelial cells), the overall stem cell enrichment is only 3-fold lower than in the MRU fractionation protocol (p = 0.049, Fig. 5B).

Using limiting cell dilutions (10k *C57Bl6* MECs) and scoring both the robust outgrowths and partial outgrowths, we confirmed that Lrp5-/cells have less stem cell activity. Interestingly, outgrowths from mixtures of control and *Lrp5−/−* cells contained labeled (control) and unlabeled Lrp5−/− cells. In other words, outgrowths were not clonal and Lrp5−/− cells can contribute to outgrowths in the presence of wild type cells (Figure S3).

Although stem cell populations are clearly depleted in Lrp5−/− glands, this has no obvious effect on function of glands during pregnancy; indeed there are no differences in lactation of multiparous Lrp5−/− breeders, or in their mammary ductal trees (Figure S4), despite at least 8 months of estrus cycling and 3 pregnancies. We conclude that regenerative basal stem cells are not required to contribute to lobuloalveolar development.

### Lrp5 Expression is Required for Maintenance of the Basal Cell Layer

**Figure 6:**
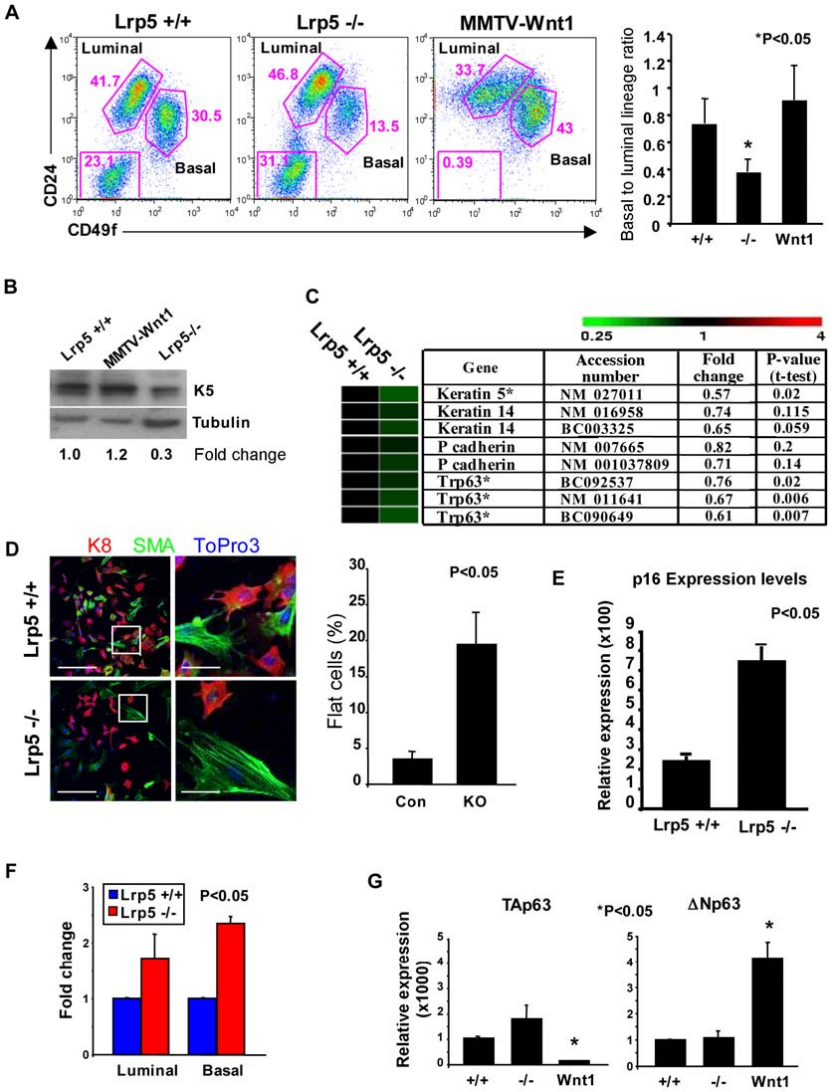
In Lrp5 Null Glands, the Basal Cell Population is Depleted. A) FACS analysis of *Lrp5* +/+, −/−, and *MMTV-Wnt1* MECs stained with CD49f and CD24. Gates were drawn to indicate the luminal and basal lineages. Right panel, quantification of the ratio of basal to luminal cells from three independent FACS analyses of CD24/CD49f profiles, shown in left panel. B) Western analysis of lysates prepared from uncultured *Lrp5* +/+, −/−, and *MMTV-Wnt1* MECs. Blots were probed with the basal cell marker, K5, and re-probed with tubulin, as a loading control. C) Representative heatmap and statistical analysis of basal markers from microarray of *Lrp5* +/+ and −/− MECs. Samples were analyzed by GeneSifter software and compared by t-test, *p<0.05. D) Immunofluorescent staining of cultured *Lrp5* +/+ and −/− MECs. Cells were cultured for 3 days in normal culture media and stained for K8 (red), SMA (green), and DNA (blue), scale bars = 20 µm. Insets depict 5×enlargements, scale bars = 4 µm. The number of large, flattened SMA positive cells were quantified from several fields of immunofluorescent staining of Lrp5 +/+ and −/− cultured MECs from two independent experiments. E) Quantification of *p16^Ink4a^* mRNA levels from *Lrp5* +/+ and −/− MECs, after 3 days of culture. F) Quantification of *p16^Ink4a^* mRNA expression levels of purified luminal and basal *Lrp5* +/+ and −/− MECs after culture. mRNA levels were normalized to the housekeeping genes, *TBP* and *HPRT*. Normalized expression = 2^−ΔCt^ (see Methods S1). G) Quantification of *TA-p63* and *ΔN-p63* mRNA expression levels of uncultured *Lrp5*+/+, −/−, and *MMTV-Wnt1* MECs. Data is expressed as fold change over Lrp5 +/+. Data were compared by Wilcoxon Rank Sum Test. Significance was established at p<0.05.

Since Lrp5 positive cells are localized to the basal epithelial layer and ductal stem cell activity is depleted in *Lrp5* null mammary glands, we quantified basal cells in populations of MECs from *Lrp5*−/− mice using flow cytometry. Though it was difficult to quantify basal cell depletion *in vivo* (Fig. 1), flow cytometric analysis revealed a two-fold depletion of basal cells with respect to total epithelial cells (Fig. 6A). The frequency of basal cells in the total mammary epithelial cell population decreased from approximately 4.2 to 2.2 basal cells per 10 epithelial cells. In contrast, basal cell frequency increased in *C57Bl6 MMTV-Wnt1* (*MMTV-Wnt1*) hyperplastic MECs, suggesting that Lrp5-dependent Wnt signaling is important for normal differentiation of basal cells.

To confirm this result, we assessed the level of expression of lineage-specific markers using biochemical analysis. Lysates were prepared from primary MECs isolated from *Lrp5* +/+, *Lrp5* −/−, and *MMTV-Wnt1* mice (to examine the loss- and gain of function conditions). There was less (0.3×) K5 expressed in *Lrp5*−/− glands, and slightly more (1.2) in glands from *MMTV-Wnt1* mice (Fig. 6B). A similar pattern was observed for K5 mRNA expression in isolated *Lrp5* −/− and *MMTV-Wnt1* MECs as well as from E10-12 embryos (Figure S5). Microarray analysis of *Lrp5* +/+ and −/− MECs further corroborated the relative depletion of cells with the basal phenotype in *Lrp5* −/− MECs, where the basal markers K5 and p63 were found to be significantly down-regulated 0.57- and 0.68-fold, respectively, compared to Lrp5+/+ MECs (Fig. 6C). In addition, analysis of markers previously characterized to define the MRU sub-population were found to be relatively down-regulated in *Lrp5* −/− MECs (Figure S6).

Analysis of *Lrp5* +/+ and −/− MECs in culture showed that a significantly higher number of morphologically abnormal basal cells appeared in *Lrp5* −/− MECs. The flattened, stressed morphology is illustrated best by staining for smooth muscle actin (SMA) (Fig. 6D). To determine whether these abnormal basal cells in *Lrp5* −/− MECs were senescent, we examined the expression of p16^Ink4a^ in cultured primary MECS. mRNA levels of p16^Ink4a^ were significantly up-regulated (3.5x) in *Lrp5* −/− MECs (Fig. 6E). To find out whether this reflected specific changes in the basal lineage, cells were purified from these populations, and the luminal and basal cells cultured independently. p16^Ink4a^ expression was significantly increased in both the basal and luminal cells of *Lrp5* −/− mammary glands (Fig. 6F).

Another basal marker that showed significantly attenuated expression in these glands is p63 (a p53 family member). Expression of this gene is under the control of two distinct promoters, and the properties of each product are functionally associated with different stages of differentiation. Thus, a full length TA-p63 isoform may regulate p53-dependent transcription and has been associated with senescent cells [36], [37]. In contrast, expression of the truncated ΔN-p63 isoform, which suppresses p53 function, is associated with proliferative cell compartments [37], [38]. mRNA isolated from MECs from *Lrp5* +/+, −/−, and *MMTV-Wnt1* mice showed higher levels of levels of *ΔN-p63* in *MMTV-Wnt1* MECs, and very low *TA-p63*. *Lrp5*−/− MECS showed a reciprocal pattern, with relatively higher *TA-p63* (Fig. 6G).

### Loss of Lrp5 does not Affect the Wnt Signaling Transactivation Response of Cultured Mammary Epithelial Cells

**Figure 7:**
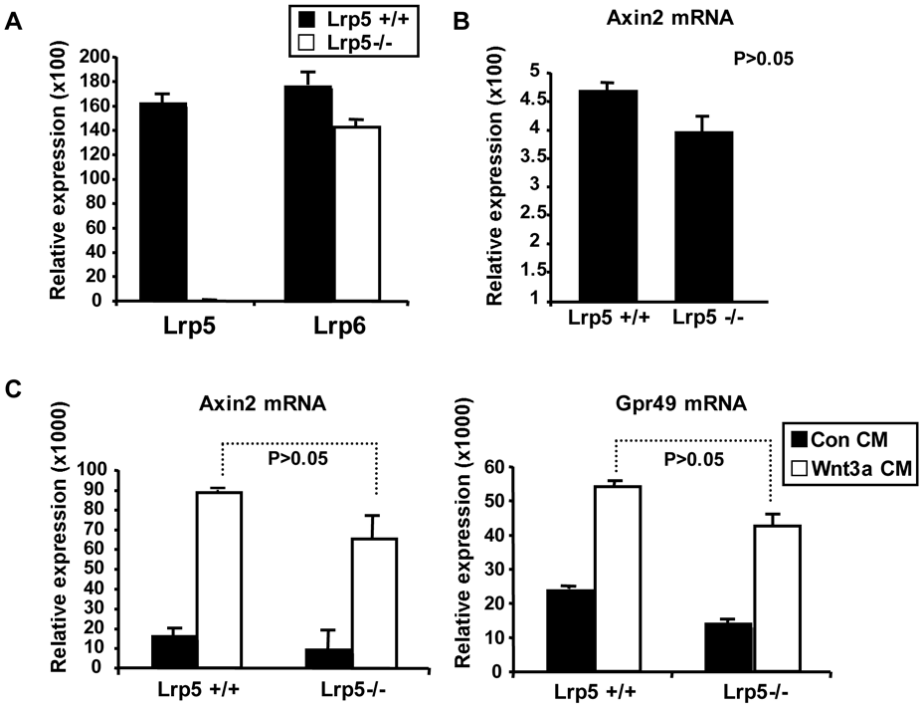
Wnt Signaling of Whole Populations is not Significantly Affected by the Absence of Lrp5. A) Quantification of *Lrp5* and *Lrp6* mRNA levels in *Lrp5* +/+ and −/− MECs. B) Quantification of expression of the Wnt target gene, *Axin2*, mRNA in *Lrp5* +/+ and −/− MECs. C) Quantification of *Axin2* and *Gpr49* mRNA expression levels in *Lrp5* +/+ and −/− MECs treated with Wnt3a or control conditioned media. All samples were cultured for three days and mRNA levels were normalized to the expression of the housekeeping genes, *TBP* and *HPRT* (as described in the Methods section). Normalized expression = 2^−ΔCt^ (see Methods S1). Data shown in A were compared by unpaired Student's t-test. Data shown in B and C were compared by Wilcoxon Rank Sum Test. Significance was established at p<0.05.

Using qPCR, we evaluated the relative expression of *Lrp5* and *Lrp6* in *Lrp5*−/− mice, to determine whether there was a compensatory expression pattern for *Lrp6* in the absence of *Lrp5*. The amount of *Lrp6* mRNA was unchanged in *Lrp5*−/− MECs from virgin *Lrp5* +/+ mice (Fig. 7A). In order to assess Wnt transactivation, we used endogenous Wnt target gene expression (to circumvent artifacts associated with transfection of highly refractive primary cells). *Axin2* and *Gpr49* are downstream target genes in the Wnt signaling pathway. In MECs from virgin mice, the amount of *Axin-2* mRNA was not affected by the absence of *Lrp5* (Fig. 7B). Cultured MECs were treated with Wnt3a-containing conditioned medium (or control), and *Axin2* was found to be induced 6×even in *Lrp5*−/− MECs. Similarly, a 2–3×induction of *Gpr49* was observed in *Lrp5*−/− MECS (Fig. 7C). Moreover, we found the maintenance of Wnt signaling in *Lrp5*−/− MECs was not due to the up-regulation of *Lrp6* in response to culture conditions, since *Lrp6* mRNA levels remained relatively unchanged throughout the culture period (Figure S7). Taken together, these results indicate that although there was a significant change in the physiology of *Lrp5*−/− glands, the canonical Wnt response was largely normal. Potential explanations for that are described in the Discussion section.

## Discussion

Previously, we have shown that Wnt1-induced, hyperplastic mammary glands accumulate undifferentiated cells (of both basal and luminal lineages) and ductal regenerative/stem cells (as a proportion of total) [9]. Here, we show an increased proportion of basal cells (measured by their CD24/CD49f profile and higher relative expression of basal marker proteins/total protein) and increased expression of a basal cell-associated p63 variant, ΔNp63, associated with proliferative function in basal cell lineages. These glands subsequently develop solitary differentiated tumors with multiple lineage characteristics. We show here (and Lindvall et al, 2006) that loss of *Lrp5* generates the reciprocal phenotype – slower ductal outgrowth, the accumulation of peri-senescent cells (of both lineages), almost total depletion of adult regenerative cells from the ductal tree, a reduced proportion of basal cells compared to luminal, and increased expression of the *TAp63* isoform, associated with senescence. We predict that these glands will be highly tumor resistant (for tumors arising in a basal cell precursor).

We have shown that Lrp5 is expressed together with Lrp6 on most/all basal cells of the mammary gland. However, most basal cells have a low cell surface expression of Lrp5, and only cells with a high level have enriched stem cell function. This fraction includes 80% of the mammary stem cells. Lrp5 is unique amongst the markers described so far for mammary epithelial populations for providing significant levels of stem cell enrichment without combining it with other markers.

Wnt1-induced mouse mammary tumors share a transcriptional signature with Brca1+/− and carcinogen-induced tumors [39], and these in turn share components of their basaloid signature with human basaloid tumors [40]. Characteristically, all of these tumors have residual basal cells, and are likely to derive from the basal lineage. It is perhaps not surprising then that the key components of the oncogenic Wnt signaling pathway are specifically expressed by basal cells.

There are data to suggest that gain of function of Lrp5 or -6 is important to breast cancer. Some human breast cancer cells have an autocrine Wnt signaling loop (Bafico et al 2004). Recently, a splice variant of Lrp5, ΔLrp5, was found in the majority (85%) of breast tumors, and was required for their continued growth. The deletion of the variant exons by splicing was associated with resistance to inhibition by Dkk1 [41]. These data suggest that ectopic Wnt signaling could be an important source of growth dysregulation in breast tumors. These tumors are not all basaloid, they include tumors of many classes, which suggests that the Lrp5-dependent growth pathway could become viable in many candidate tumor precursor cell types.

Lrp5 and -6 are co-expressed in the majority of basal cells. Lrp6 co-expression is high enough that the absence of Lrp5 has no effect on Wnt transactivation in response to Wnt3A (with no gross transcriptional compensation of Lrp6 mRNA). However, there is a very specific effect of the loss of *Lrp5* on the maintenance of adult somatic stem cell activity. This corresponds to the lack of tumorigenicity of Wnt1 in *Lrp5*−/− mammary glands (Lindvall et al, 2006). This could be explained by the following scenarios - 1) Lrp5 has a distinct function in mammary stem cell biology compared to Lrp6, 2) Lrp5 expression augments Lrp6 expression to push the total expression over a critical threshold for growth promotion, 3) both Lrp5 and -6 are required for the stem cell function, 4) the ligand for Lrp5/Fzd is not Wnt1/Wnt3A (whereas Wnt3A binds and stimulates Lrp6/Fzd), or 4) Lrp5 has a different subcellular presentation from Lrp6.

Often, Lrp5/6 are used in experiments interchangeably, since they exhibit high sequence homology. Though they show similar expression patterns in embryonic and adult tissues [42], [43], [44], they have distinct functions. For example, an allelic series of mutations in *Lrp5* and *6* in mouse embryos revealed that the *Lrp5* loss in combination with *Lrp6* loss produces a more severe phenotype than *Lrp6* loss alone, and that *Lrp6* loss is more severe than *Lrp5* loss alone [22], [45]. The loss of *Lrp5* tends to produce a subset of the phenotypes typical of *Lrp6* null mice. One possibility is relative ligand specificity, and this may be influenced by the presence of LDLR repeat sequences in the extracellular domains. Although the extracellular domains are highly conserved between the two receptors (73%), the LDLR repeats demonstrate significantly lower sequence homology [44]. In addition, the extraceullar domain of Lrp6 contains 10 putative glycosylation sites, whereas Lrp5 only contains 5, which may also play a role in directing ligand specificity.

Another possible mechanism leading to the divergent functions of Lrp5 and 6 may be cell surface presentation and intracellular processing of the receptors. Internalization of Lrp6 has been found to be regulated by clathrin, caveolin, ligand interaction (including R-spondin) and γ-secretase-dependent mechanisms [46], [47], [48], [49], [50], indicating Lrp6 (and possibly Lrp5) activity can be regulated by different internalization patterns. Although the internalization mechanism of Lrp5 is unknown, it likely differs from that of Lrp6, since the intracellular domains are much less conserved [44].

It is likely that Wnt ligands have specific cognate Frizzled receptors. This specificity has been demonstrated for Wnt5A, which can induce canonical signaling only when Fzd4 and Lrp5 (not Lrp6) are present [51]. Wnt5A is known to be expressed and bioactive during mouse mammary gland development; a gain of function inhibits ductal outgrowth, and loss promotes hyperproliferation [52]. Most Fzd mRNAs are expressed in virgin ductal mammary glands, except for Fzd4. If Fzd4 is expressed, it is a very low level, or in a very small subpopulation (unpublished data, Y C Kim and C M Alexander).

The overall output of canonical and other responses depends upon the relative amount of receptors and ligands. Non-canonical and canonical pathways can inhibit each other, and even non-productive interactions (for example Wnt5A:Lrp6) can compete with normal signaling activities (for example, activation of the non-canonical target Rac1) [53], [54]. It is possible that Lrp5 binds Wnt5A during normal development (with no canonical signaling effect), but the absence of Lrp5 enhances the inhibitory effects of Wnt5A on ductal outgrowth (and more specifically, stem cell self-renewal).

Although the embryonic outgrowth of mammary rudiments is Wnt-dependent [15], [16], the phenotype of early mammary development of *Lrp5*−/− mice is only marginally affected. This suggests that only the adult somatic ductal stem cells require Lrp5, for either 1) stem cell specification during tree outgrowth, or 2) survival in adult glands. Prior modeling has predicted that if most cells in the mammary rudiment have at least 30 division cycles, there is a reserve of growth potential [55]. In fact, the challenge of development appears to be to control and attenuate the growth potential to enable functional differentiation. In other words, there is no need to invoke stem cells to explain the growth associated with ductal outgrowth, estrus cycling, or pregnancy. The *Lrp5* null mouse is an example of this, as is the *β1 integrin* null mouse [56], [57], [58]. Both these strains show approximately normal ductal extension, but neither ductal tree has significant regenerative capacity. Yet, we report that the stem cell-deficient gland is affected in a predictable way. When mammary epithelial cell populations are transferred to culture, there is increased expression of senescence-associated markers, such as p16^Ink4a^ and TAp63. (Our data suggests that senescence markers are transient *in vivo*, unless glands are actively growing; presumably cells are removed by apoptosis or autophagy). By separating the luminal and basal cells for independent culture, we show that the effect of the Lrp5 null mutation is evident not only in the basal cell population (where we would anticipate the effect of this mutation), but also in the luminal cells. We propose that this is consistent with the stem cell origin of this effect.

Cellular senescence is described as a natural mechanism of tumor suppression [59]. The mechanism of several tumor suppressors has been demonstrated to be the induction of senescence or apoptosis. More specifically, it has been proposed that tumor suppressors may act by reducing the stem/progenitor cell pool, since overexpression often leads to a reduction in the regenerative capacity of a tissue [59]. For example, the tumor suppressor, p16^Ink4a^, is thought to act this way. It is deleted or inactivated in numerous tumors, whereas overexpression results in senescence and an aged phenotype [60]. Indeed, ectopic p16^Ink4a^ expression has been shown to deplete stem cell activity in a number of tissues (brain, pancreas, and hematopoetic system) [61], [62].

Similar to p16^Ink4a^, p53, is also a widely recognized tumor suppressor, where loss of function mutations are associated with tumorigenesis and gain of function mutations result in aging and senescence [59]. p63 is a closely related family member to p53, yet very little is known about the function of this protein. It has been shown to be required for mammary gland development [63] and is frequently up-regulated (although, not mutated) in several epithelial cancers [38]. The TAp63 isoform has been shown to be pro-apoptotic [64] and can bind to p53 response elements, driving transcription of p53 target genes. The ΔNp63 isoform, however, acts as a dominant-negative competitor for TAp63 and p53 [65]. The ΔNp63 isoform is expressed at higher levels than TAp63 during development and at lower levels during differentiation [66], [67]. Consequently, it has been suggested that the ratio of ΔNp63 to TAp63 isoform expression may dictate whether a cell follows its normal differentiation program, becomes senescent, or undergoes oncogenic transformation [38]. It is, therefore, not surprising that ΔNp63 is the predominant isoform expressed in human breast cancers [38]. Interestingly, ΔNp63 has been shown to interact with and regulate the Wnt signaling pathway, promoting cell proliferation [68]. Thus, Wnt signaling through Lrp5 may regulate the proliferative potential of the basal mammary stem cell population by inhibiting senescence (as it does in various human cell lines *in vitro*, [69]). We conclude that profound differences in regenerative potential are not necessarily reflected at the gross level of epithelial organogenesis. Instead, there are changes in the predisposition of the cellular populations to senescence, and perhaps to growth stimuli and transforming events.

## Materials and Methods

### Mice and Materials

*C57Bl6 Lrp5* +/+, −/− [29], [70], *C57Bl6 MMTV-Wnt1* [71], *C57BL/6-Tg(CAG-EGFP)1Osb/J* (strain 003291, ubiquitous EGFP expression, driven by β-actin promoter) and *BALB/c* (Jackson Labs, Bar Harbor, ME) mice were bred and maintained in accordance with the guidelines set forth by the National Institutes of Health Guide for the Care and Use of Laboratory Animals, published by the U.S. Public Health Service. All experimental protocols were approved by the Institutional Animal Care and Use Committee at the University of Wisconsin-Madison. Fluorescent conjugated rat monoclonal antibodies against CD49f (GoH3), CD24 (M1/69), CD45 (30-F11), and CD31 (MEC 13.3) were purchased from BD Biosciences (San Jose, CA). Mouse anti-Lrp6 was generated as previously described [46] and Lrp5 (41–130) was purchased from Abnova (Taipei City, Taiwan). Mouse monoclonal antibodies against SMA (1A4), tubulin (JDR.3B8), and actin (AC-15) were purchased from Sigma Aldrich (St. Louis, MO). Rabbit anti-K5 was purchased from Covance (Madison, WI), rabbit-anti VWB was purchased from Dako (Glostrup, Denmark), and rat anti-K8 (Troma-I) was purchased from the Developmental Studies Hybridoma Bank (University of Iowa). All fluorescent conjugated secondary antibodies were purchased from Molecular Probes (Eugene, OR).

### MEC Isolation and Culture

MECs were prepared from mouse mammary glands as previously described [34], using reagents and protocol from Stem Cell Technologies (Vancouver, CA). Briefly, mammary glands were isolated from virgin or 14 day pregnant 12–14 week old *C57Bl6 Lrp5* +/+, −/−, *MMTV-Wnt1*, or *BALB/c* virgin mice. The mammary glands were cut into small pieces with fine scissors and digested for 6 hours, 37°C in Epicult-B supplemented with 5% fetal bovine serum, 300 U/mL collagenase and 100 U/mL hyaluronidase. The resulting mammary organoids were washed, counted, and frozen in DMEM containing 10% FBS and 10% DMSO until further use. Prior to cell culture, mammary organoids were reduced to single cells by digesting in consecutive one-minute incubations with trypsin and dispase. The cells were passed through a 70 µm mesh filter prior to seeding into Matrigel-coated plates [72]. Cells were maintained in either normal culture media (DMEM supplemented with 10% FBS, 10 µg/ml insulin, and 100 U/m Pennicillin/Streptomycin) or L-Cell (Con) or Wnt-3a (Wnt) conditioned media [73] diluted 50% with normal culture media.

### Northern Blotting

*Lrp5* +/+, +/−, and −/− embryos were isolated from approximately 18 day pregnant mice, flash frozen in liquid nitrogen, and transferred to RNAlater -ICE (Ambion, Foster City, CA) before RNA isolation. A similar strategy was used for isolated MECs and cell lines (RNAlater (Ambion)/RNeasy mini kit; Qiagen). NMuMG cells were cultured in DMEM supplemented with 10% FBS and 10 µg/mL insulin (Sigma). HC11 cells were cultured in RPMI 1640 supplemented with 10% FBS, 5 µg/mL insulin, and 10 ng/mL rEGF (R&D Systems, Minneapolis, MN). The preparation of Northern blots and probes is described in Methods S1.

### Western Blotting

Embryos isolated from 10–12 day pregnant *C57Bl6 Lrp5* +/+ or Lrp5 −/− mice were homogenized for 1 min/4°C in “Streuli” lysis buffer [74] using a Polytron homogenizer. Cultured cells were harvested by scraping into culture medium, centrifuged at 180×g for 5 min, resuspended in lysis buffer, and incubated for 30 minutes on ice. Fresh, purified mammary epithelial cells were quickly thawed, washed with culture media, resuspended in lysis buffer, and incubated for 30 min on ice. All lysates were cleared at 12,000×g for 30 min. Supernatants were removed and protein concentration determined (using Bradford reagent (Pierce, Rockford, IL)) and analyzed by SDS-PAGE and immunoblotting (as previously described [72]).

### Immuofluorescence

Mammary tissue sections were prepared from either virgin or 10–12 day pregnant, adult female *Lrp5* +/+ and *Lrp5* −/− mice. Virgin mammary glands were fixed overnight in 2% paraformaldehyde at 4°C, embedded in paraffin, and sectioned (8 µm). Tissues were deparaffinized, re-hydrated, and blocked with 10% non-immune goat serum for 1 h at room temperature. Pregnant mammary glands were frozen in OCT compound (Electron Microscopy Sciences, Hatfield, PA) prior to sectioning. Frozen sections were fixed in acetone for 20 min at room temperature prior to blocking with 10% non-immune goat serum for 1 h and overnight incubation with anti-Lrp5, K5, SMA, or K8 antibodies. Samples were incubated with anti-mouse IgG-Alexa 488 and anti-rabbit or rat IgG-Alexa 546 secondary antibodies for 2 h at room temperature. Nuclei were stained by incubation with ToPro3 (Molecular Probes) for 30 min prior to visualization using confocal microscopy (Radiance 2100, Biorad, Hercules, CA). For “cytosplats,” single cell suspensions of FACS sorted mammary epithelial cell populations were dried briefly onto microscope slides and fixed in ice-cold methanol and acetone for 4 and 2 minutes, respectively. Cell preparations were blocked in 10% non-immune goat serum and stained as above. The number of K5 and K8 positive cells were quantified by manual counting of at least 100 cells in three random fields per experimental sample.

### Flow Cytometry

Single cell suspensions of *BALB/c* mammary epithelial cells were prepared from frozen preparations of mammary organoids, as described above. The resulting single cell suspensions were stained with Lrp5 or Lrp6 in addition to fluorescent conjugated rat monoclonal antibodies to CD49f, CD24, CD45, and CD31 for 30 min on ice. The cells were washed and incubated with anti-mouse IgG-Alexa 405 for 30 min on ice, and analyzed on a FACSVantage cell sorter with DiVa software (Becton Dickson, Franklin Lakes, NJ). Live cells were discriminated by propidium iodide exclusion. CD45 and CD31 positive cells were excluded prior to gating of populations based on Lrp5 expression levels. For functional evaluation, cell populations which exhibited high, low, or negative Lrp5 staining, together with a CD24^+^CD49f^high^ (MRU) fraction for comparison, were sorted into pure fetal bovine serum, centrifuged at 700×g for 5 min, re-suspended in culture media containing 10% DMSO, and frozen in liquid nitrogen prior to transfer into recipient mice. To identify the population of Lrp5 high expressing cells that were not localized to the MRU sub-population, virgin *BALB/c* MECs were stained with anti-VWB in addition to CD49f and CD24.

### Microarray

MECs were isolated from 6 groups of *Lrp5*+/+ and 3 groups of *Lrp5*−/− mice, as described above. Each sample group comprised 5 mice each that spanned 12–16 weeks of age. Estrous staging was performed on the mice by vaginal cytological examination and mice belonging to different stages of the cycle were assigned to each group randomly. Total RNA was isolated from MECs using the Qiagen RNeasy kit (Qiagen), as described above. The isolated RNA was submitted to the Gene Expression Center, University of Wisconsin-Madison where a quality control test was performed on the RNA samples. RNA from each group was used for cDNA synthesis followed by labeling of the cDNA with Cy3. The labeled cDNA samples were submitted to NimbleGen and hybridized to Mus musculus 1-Plex arrays (Roche NimbleGen, A4543-00-01) that represent 42,586 mouse genes. The single color NimbleGen arrays were scanned with a GenePix 4000B microarray scanner. The data was extracted from scanned images using NimbleScan software and the Robust Multichip Average (RMA) algorithm used to generate gene expression values. The details of labeling, hybridization, scanning and normalization of the data are described in detail on the NimbleGen website (http://www.nimblegen.com). The normalized data was subsequently analyzed using GeneSifter, an online microarray data analysis system (http://www.genesifter.net). Heat maps were generated from pooled *Lrp5* +/+ and −/− samples. T-tests were used to determine the statistical significance between the two groups which was established at p<0.05.

### Quantitative RT-PCR

*Lrp5* +/+ and −/− MECs from 12–14 week old mice were cultured on matrigel-coated plates and treated with Wnt-3a (Wnt) or L Cell (Con) conditioned media for 16 h, 1, 2, or 3 days. Total RNA was isolated using the RNeasy Mini Kit (Qiagen) according to manufacturer instructions. cDNA was generated using a mix of oligo dT and random primers using QuantiTect Reverse Transcription Kit (Qiagen). cDNA (100 ng) was amplified by real time PCR using 5 µL SYBR Green qPCR SuperMix-UDG with Rox (Invitrogen) and 4 µL of forward and reverse primers (0.5 µM). The analysis was performed on each sample in triplicate with an ABI 7900-HT (Applied Biosystems, Foster City, CA). Relative transcript levels were calculated using the comparative Ct method and normalized to the previously characterized housekeeping genes, tata binding protein (*TBP*) and hypoxanthine-guanine phosphoribosyltransferase (*HPRT*) [75]. Primer sequences and additional methods are described in Methods S1.

### Mammary Reconstitution Assay

Mammary glands of 3-week old *BALB/c* virgin mice were cleared of endogenous epithelium as previously described [76]. Populations of mammary epithelial cells isolated by flow cytometry were quickly thawed, washed, counted, and re-suspended in DMEM containing 5 µg/mL Matrigel and loading dye (5% glycerol/0.5% trypan blue/25 mM HEPES pH 7.2). A 1 µL volume of cell suspension containing 50–50,000 cells was injected into each cleared fat pad of the recipient mice. The fat pads were removed 8 weeks following transplants, fixed in 2% PFA overnight at 4°C, stained with carmine alum, dehydrated in ethanol, and de-fatted in xylene. Outgrowths were subsequently identified using a light microscope, photographed, and the number of outgrowths quantified for each dose of cells transplanted.

### Estimation of Stem Cell Frequencies and Statistical Analyses

Stem cell frequencies were estimated using the limdil software for limiting dilution analysis (http://bioinf.wehi.edu.au/software/limdil). All other data were compared by either unpaired Student's T-test or Wilcoxon Sum Rank test, using Mstat 4.0 statistical software (Norman Drinkwater, McArdle Laboratory). Significance was established at p<0.05.

## Supporting Information

Figure S1 Evaluation of Antibody Specificity. To determine whether there is any cross-reactivity between the Lrp5 and Lrp6 antibodies, 293 cells were transfected with plasmids encoding Lrp5 or Lrp6. A) Western blot of transfected 293 cell lysates for Lrp5 and Lrp6. Actin was used as a loading control. B) Immunofluorescence of transfected 293 cells stained for either Lrp5 or Lrp6 (red). Nuclei were stained with ToPro3 (blue). C) FACS analysis of transfected 293 cells. Gates were set based on staining levels seen in the untransfected control samples. Scale bar = 50 µm.
(3.84 MB TIF)

Figure S2 Specificity of Lrp5 Antibody Staining of MECs by FACS. A) C57Bl6 Lrp5 +/+ MECs were isolated, reduced to single cell suspensions, and stained with anti-Lrp5, in addition to CD24 and CD49f (and Lin antisera) prior to FACS analysis. B) C57Bl6 Lrp5 −/− MECs were isolated and stained as described in A. Top Panels: gating strategy for exclusion of cell doublets, apoptotic (PI+), hematopoetic (CD45+), and endothelial (CD31+) cells. Bottom Panels: Gates were drawn for Lrp5 expression on the basis of the staining pattern for the Lrp5−/− MEC population. Lrp5 positive (blue) and Lrp5 negative (red) populations were overlaid on the CD24/CD49f staining profile.
(3.01 MB TIF)

Figure S3 Ductal Outgrowth of Lrp5 −/− MECs is Enhanced by the Presence of GFP Labeled Control Cells. MECs were isolated from C56Bl6 Lrp5 −/− and Actin-GFP mice (12–14 weeks old). They were counted and mixed in various proportions to total 10,000 cells, and then tested for their relative reconstitution activity after transfer into cleared fat pads of C57Bl6 Lrp5 +/+ mice. Outgrowths were analyzed for the presence of GFP positive cells, and subsequently Carmine stained. They are presented as % colonization of glands, and % of each outgrowth that is GFP+ (black = proportion of unlabeled cells; green = proportion of GFP+ cells).
(1.33 MB TIF)

Figure S4 The Ductal Stem Cell Deficiency Observed in Lrp5−/− Glands does Not Affect Ductal Integrity after Multiple Rounds of Parity. Control (A) and Lrp5−/− (B) glands were evaluated by whole mount staining, and show similar structure and fat pad colonization. Scale bar = 1 mm.
(3.89 MB TIF)

Figure S5 K5 mRNA Expression is Decreased in Lrp5 −/− MECs and Embryos. A) Quantification of K5 mRNA expression in uncultured Lrp5 +/+, −/−, and MMTV-Wnt1 MECs. B) Quantification of K8 mRNA expression in Lrp5 +/+, −/−, and MMTV-Wnt1 MECs. C) Quantification of K5 mRNA expression in E10-12 Lrp5 +/+, +/−, and −/− embryos. D) Quantification of K8 mRNA expression in E10-12 Lrp5 +/+, +/−, and −/− embryos. All data were normalized to the housekeeping genes TBP and HPRT.
(0.41 MB TIF)

Figure S6 Lrp5 −/− MECs are Depleted in Markers Expressed by MRUs. Representative heatmap and stastical analysis microarray of Lrp5 +/+ and −/− RNA samples. Genes previously characterized to be up-regulated in MRUs (Stingl et al, 2006) were compared for each sample using GeneSifter software. Data were compared by student's t-test, *p<0.05.
(0.96 MB TIF)

Figure S7 Cell Culture Does not Alter Lrp5/6 Expression. A) RNA from cultured Lrp5 +/+ and −/− MECs was isolated from cells daily, and quantitative RT-PCR was performed for Lrp5 (A) and Lrp6 (B). Expression levels were normalized for each sample using the housekeeping genes, TBP and HPRT.
(0.40 MB TIF)

Methods S1 (0.03 MB DOC)
