# Supplementary figures and images for: The Wnt Receptor, Lrp5, Is Expressed by Mouse Mammary Stem Cells and Is Required to Maintain the Basal Lineage
Source: PLoS One. 2009 Aug 12;4(8):e6594. doi: 10.1371/journal.pone.0006594 (PMC2720450; doi:10.1371/journal.pone.0006594)

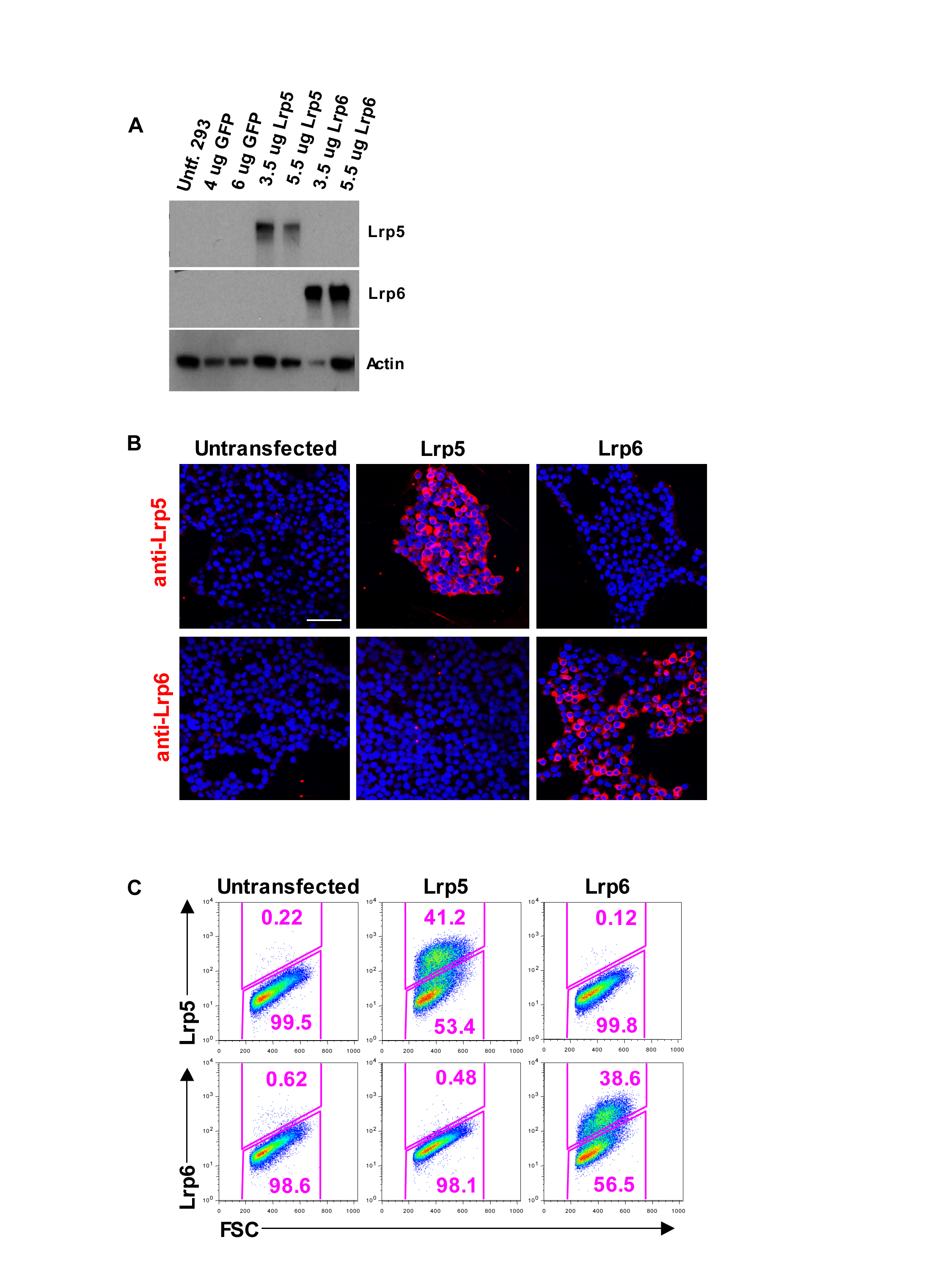

Supplement: Figure S1 — Evaluation of Antibody Specificity. To determine whether there is any cross-reactivity between the Lrp5 and Lrp6 antibodies, 293 cells were transfected with plasmids encoding Lrp5 or Lrp6. A) Western blot of transfected 293 cell lysates for Lrp5 and Lrp6. Actin was used as a loading control. B) Immunofluorescence of transfected 293 cells stained for either Lrp5 or Lrp6 (red). Nuclei were stained with ToPro3 (blue). C) FACS analysis of transfected 293 cells. Gates were set based on staining levels seen in the untransfected control samples. Scale bar = 50 µm. (3.84 MB TIF) [file pone.0006594.s001.tif]

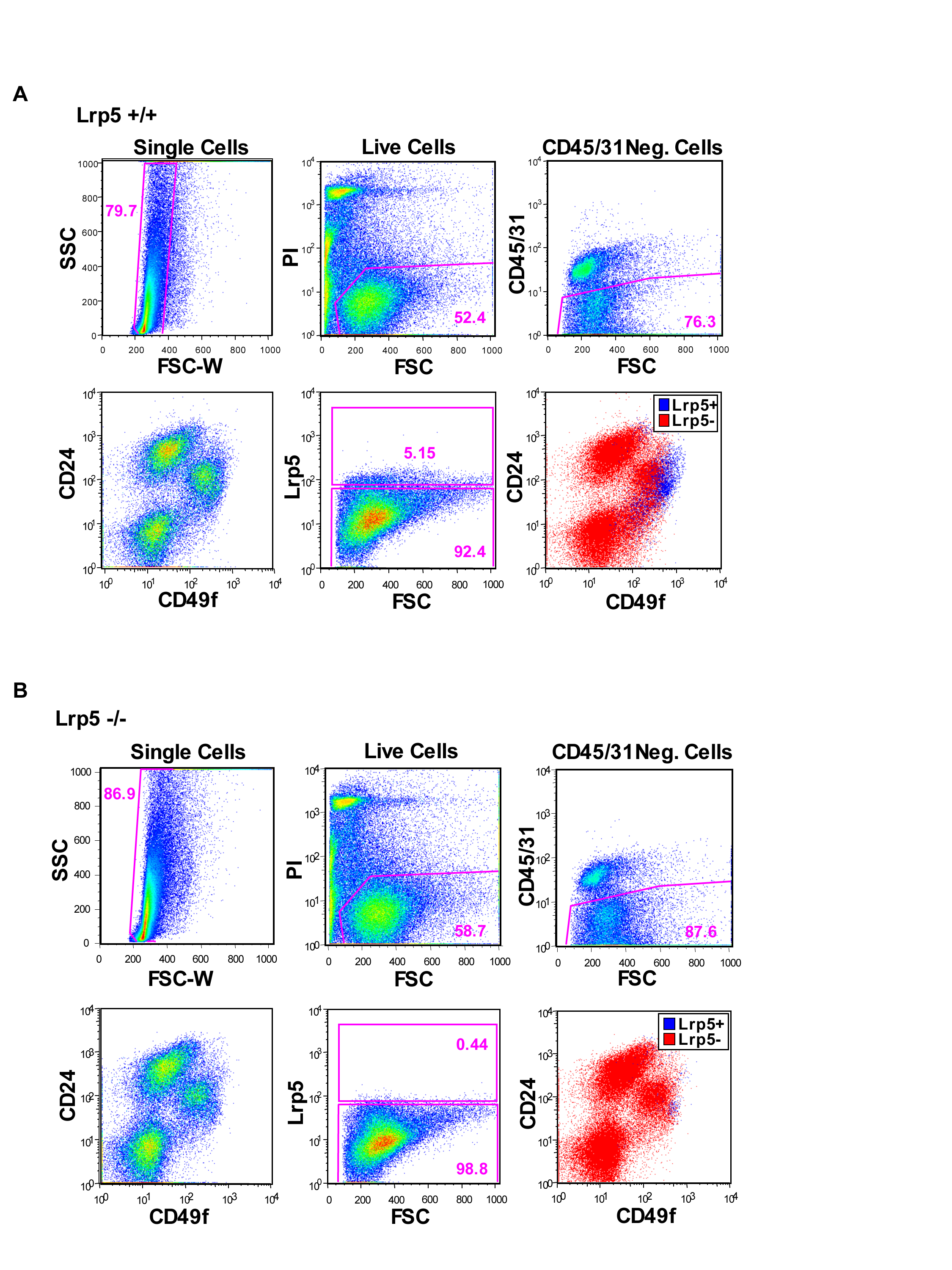

Supplement: Figure S2 — Specificity of Lrp5 Antibody Staining of MECs by FACS. A) C57Bl6 Lrp5 +/+ MECs were isolated, reduced to single cell suspensions, and stained with anti-Lrp5, in addition to CD24 and CD49f (and Lin antisera) prior to FACS analysis. B) C57Bl6 Lrp5 −/− MECs were isolated and stained as described in A. Top Panels: gating strategy for exclusion of cell doublets, apoptotic (PI+), hematopoetic (CD45+), and endothelial (CD31+) cells. Bottom Panels: Gates were drawn for Lrp5 expression on the basis of the staining pattern for the Lrp5−/− MEC population. Lrp5 positive (blue) and Lrp5 negative (red) populations were overlaid on the CD24/CD49f staining profile. (3.01 MB TIF) [file pone.0006594.s002.tif]

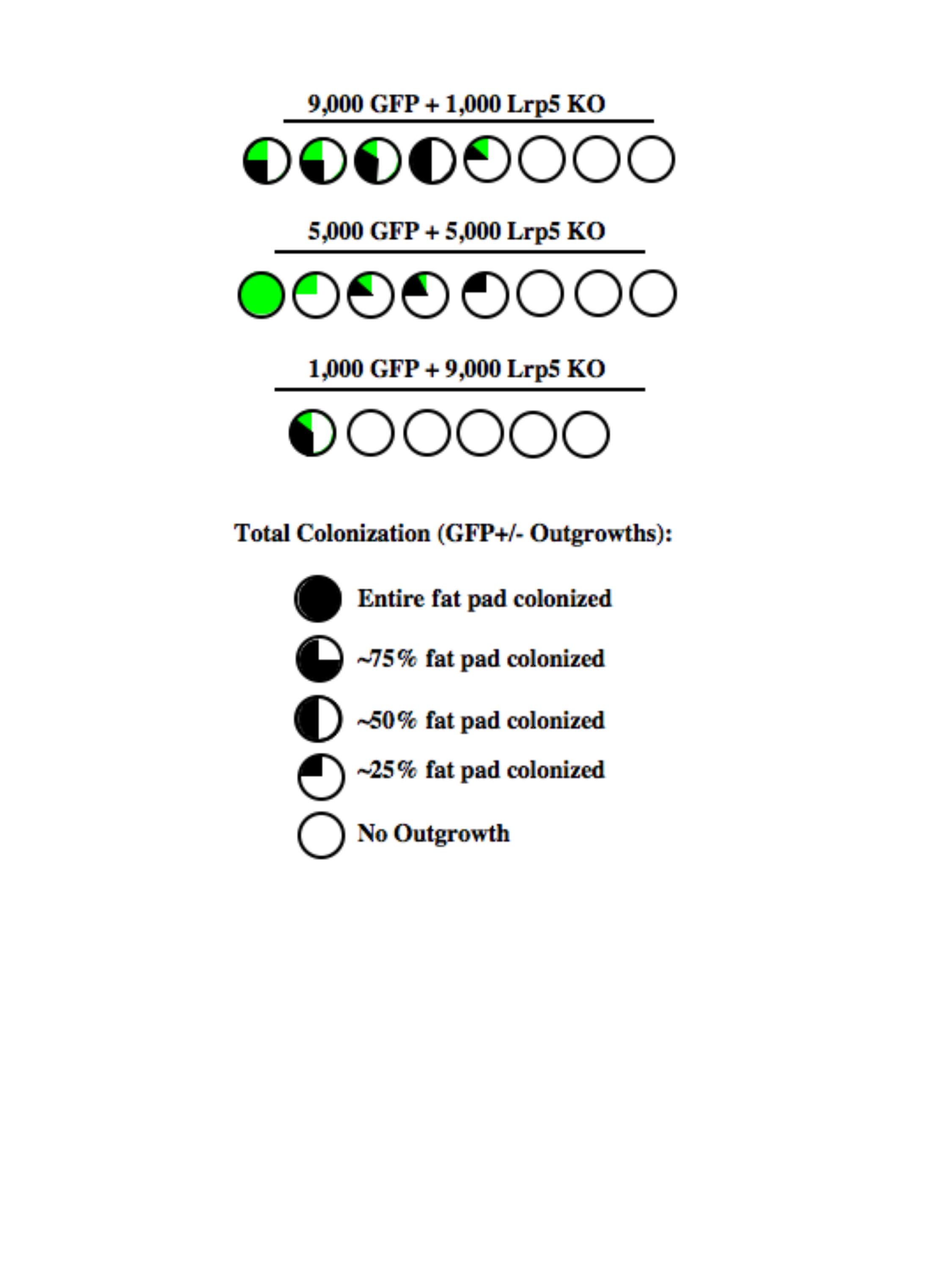

Supplement: Figure S3 — Ductal Outgrowth of Lrp5 −/− MECs is Enhanced by the Presence of GFP Labeled Control Cells. MECs were isolated from C56Bl6 Lrp5 −/− and Actin-GFP mice (12–14 weeks old). They were counted and mixed in various proportions to total 10,000 cells, and then tested for their relative reconstitution activity after transfer into cleared fat pads of C57Bl6 Lrp5 +/+ mice. Outgrowths were analyzed for the presence of GFP positive cells, and subsequently Carmine stained. They are presented as % colonization of glands, and % of each outgrowth that is GFP+ (black = proportion of unlabeled cells; green = proportion of GFP+ cells). (1.33 MB TIF) [file pone.0006594.s003.tif]

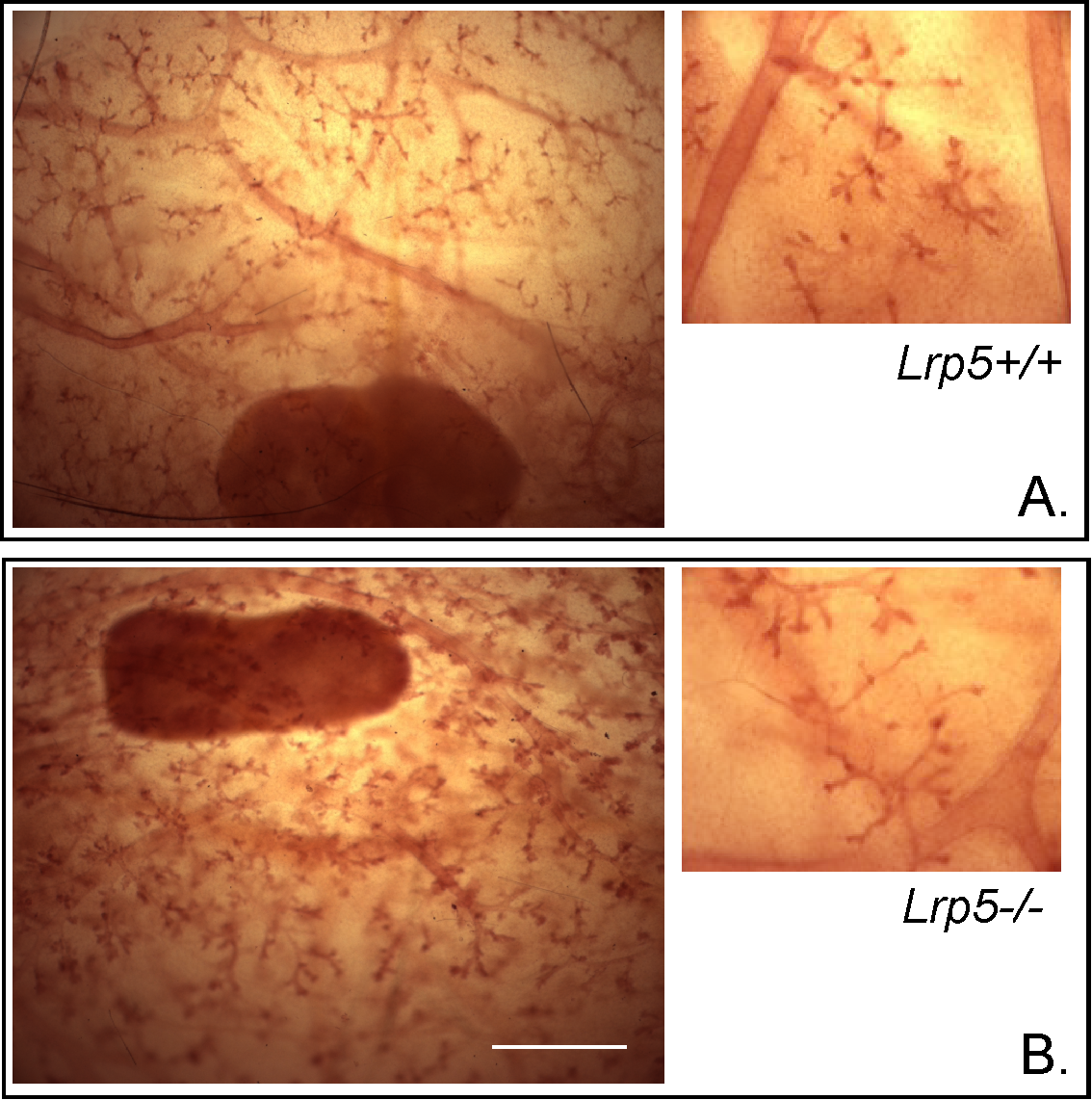

Supplement: Figure S4 — The Ductal Stem Cell Deficiency Observed in Lrp5−/− Glands does Not Affect Ductal Integrity after Multiple Rounds of Parity. Control (A) and Lrp5−/− (B) glands were evaluated by whole mount staining, and show similar structure and fat pad colonization. Scale bar = 1 mm. (3.89 MB TIF) [file pone.0006594.s004.tif]

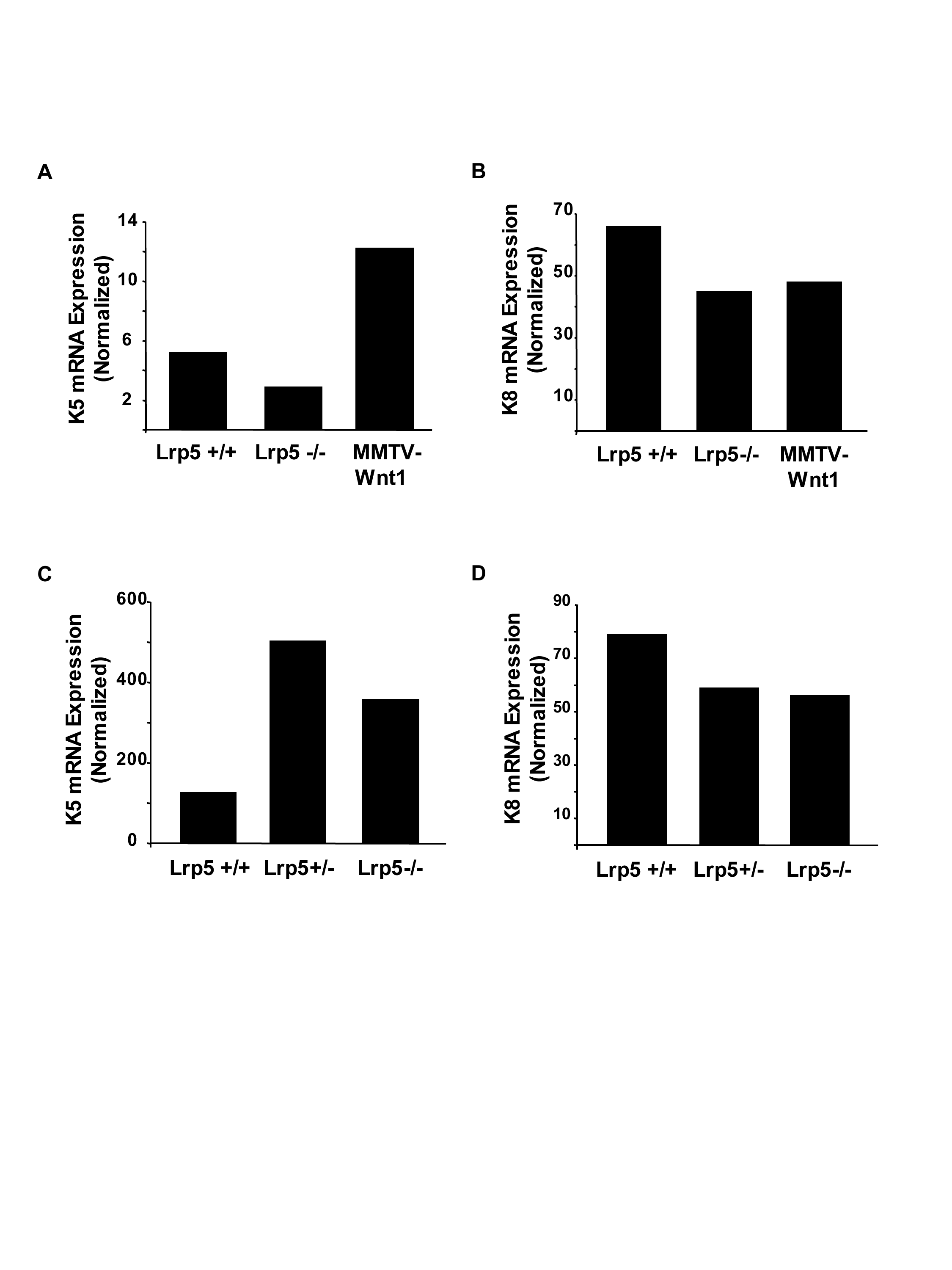

Supplement: Figure S5 — K5 mRNA Expression is Decreased in Lrp5 −/− MECs and Embryos. A) Quantification of K5 mRNA expression in uncultured Lrp5 +/+, −/−, and MMTV-Wnt1 MECs. B) Quantification of K8 mRNA expression in Lrp5 +/+, −/−, and MMTV-Wnt1 MECs. C) Quantification of K5 mRNA expression in E10-12 Lrp5 +/+, +/−, and −/− embryos. D) Quantification of K8 mRNA expression in E10-12 Lrp5 +/+, +/−, and −/− embryos. All data were normalized to the housekeeping genes TBP and HPRT. (0.41 MB TIF) [file pone.0006594.s005.tif]

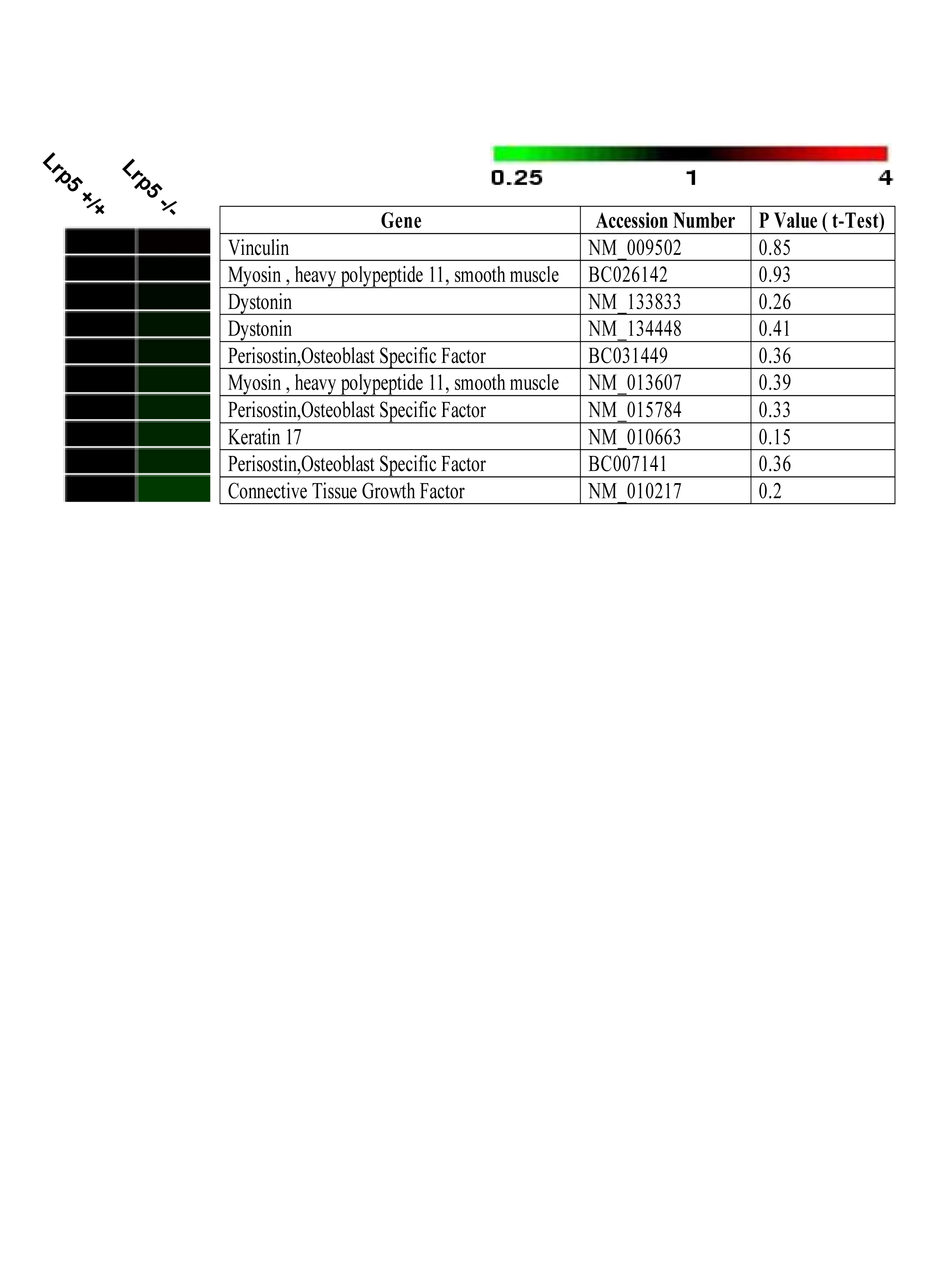

Supplement: Figure S6 — Lrp5 −/− MECs are Depleted in Markers Expressed by MRUs. Representative heatmap and stastical analysis microarray of Lrp5 +/+ and −/− RNA samples. Genes previously characterized to be up-regulated in MRUs (Stingl et al, 2006) were compared for each sample using GeneSifter software. Data were compared by student's t-test, *p<0.05. (0.96 MB TIF) [file pone.0006594.s006.tif]

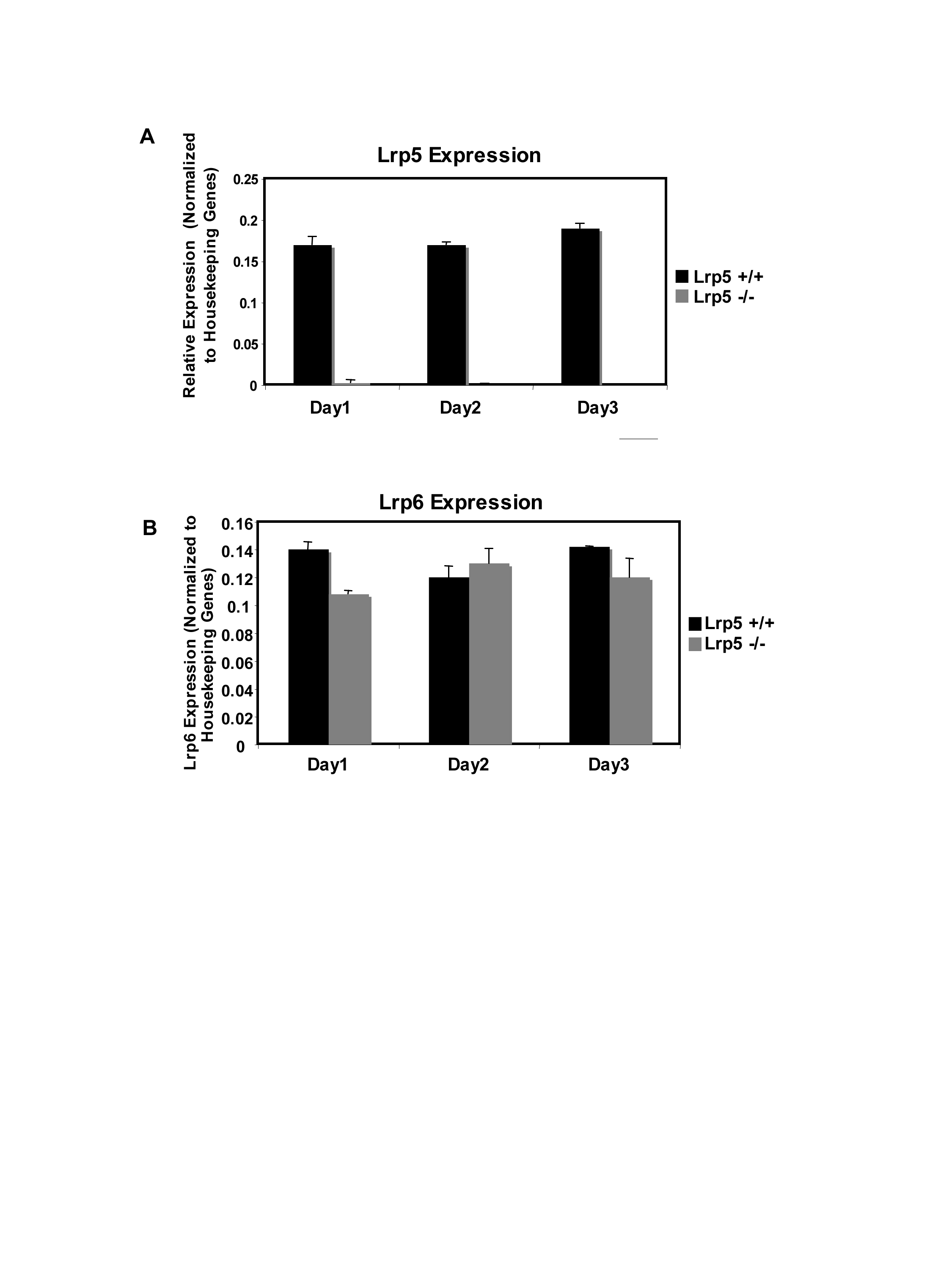

Supplement: Figure S7 — Cell Culture Does not Alter Lrp5/6 Expression. A) RNA from cultured Lrp5 +/+ and −/− MECs was isolated from cells daily, and quantitative RT-PCR was performed for Lrp5 (A) and Lrp6 (B). Expression levels were normalized for each sample using the housekeeping genes, TBP and HPRT. (0.40 MB TIF) [file pone.0006594.s007.tif]
