# Supplementary material for: The Wnt Receptor, Lrp5, Is Expressed by Mouse Mammary Stem Cells and Is Required to Maintain the Basal Lineage
Source: PLoS One. 2009 Aug 12;4(8):e6594. doi: 10.1371/journal.pone.0006594 (PMC2720450; doi:10.1371/journal.pone.0006594)
Supplement: Methods S1 — (0.03 MB DOC) [file pone.0006594.s008.doc]

**Methods:**

***RT-PCR Primer Sequences-*** The 5’ to 3’ sequences of the primer pairs used are as follows:

TBP forward: AGAATAAGAGAGCCACGGAC

TBP reverse: CAACTTCTGCACAACTCTAGC

HPRT forward: TTATCAGACTGAAGAGCTACTGTAATG

HPRT reverse: TTACCAGTGTCAATTATATCTTCAACAATC

Axin2 forward: TTTGGCACAGCTAGAGGAAG

Axin2 reverse: TGGCTCTTTGTGATCTTCTGG

Gpr49 forward: CCTTCACAGCCTCAAAGTG

Gpr49 reverse: GCAGGGATTGAAGGCTTCTC

P16 forward: CCCAACGCCCCGAACT

P16 reverse : GCAGAAGAGCTGCTACGTGAA

Lrp5 forward: AAGCAACAGTGTGACTCCTTC

Lrp5 reverse: GGGAGAGGATGATACCAATGAC

Lrp6 forward : GGTCTCACCATCGACTATGC

Lrp6 reverse: AAGGATGAGGCAAGTCATCTG

***Northern Probes-*** To generate Northern probes, *LRP5* was excised from pCMV-Sport 6 with the restriction enzymes NotI, EcoRV, and XmnI (New England Biolabs). *LRP6* was excised from pYX-Asc with NotI. Both products were run on 1% agarose gels and purified with QIAquick Gel Extraction Kit (Qiagen). Probes were created using 50 ng of each gene as a template with the Strip-EZ DNA Kit (Ambion) according to the manufacturers instructions.

***Transfections-*** Plasmid DNA was isolated from the Mammalian Gene Collection clone #3154246 (Open BioSystems) and clone #6409058 (Invitrogen), using the QIAfilter Pasmid Maxi Kit (Qiagen) according to the manufacturers protocol. The *LRP5* gene, clone #3154246, in pCMV-Sport was ready for eukaryotic expression. However, the *LRP6* gene, clone #6409058, in pYX-Asc was excised from the original vector with the restriction enzyme NotI (New England Biolabs) and purified with QIAquick Gel Extraction Kit (Qiagen). A eukaryotic expression vector, pcDNA3, (Invitrogen), was linearized with NotI. The two were ligated at room temperature for 1 hour with T4 DNA Ligase (New England Biolabs) according to the manufacturer instructions. E. *coli* DH5a (Invitrogen) were transformed with 1 μl of ligation mix. Ten colonies were prepped with QIAprep Spin Miniprep Kit (Qiagen) according to manufacturer instructions. A restriction digest with BamHI and HindIII (New England Biolabs) enzymes was used to screen for properly ligated clones. 293 cells were grown to near confluency in a 6 well plate and transfected with *LRP5* (Open Biosystems) and *LRP6* (Invitrogen) clones using Lipofectamine (Invitrogen) according to the manufacturer instructions. Briefly, 6, 4.5, 3, and 2 μg of plasmid DNA were added to individual wells. For visual indication of successful transfection, 0.5 μg of PcmmP eGFP DNA (Liu et all, 2003) was included in each well.

***FACS Staining/Analysis-*** Primary, uncultured MECs were isolated from 12 week old, virgin *C57Bl6* *Lrp5* +/+ and -/- mice. The MECs were reduced to single cell suspensions using reagents and protocol from Stem Cell Technologies prior to staining. The cells were stained with the following rat antibodies: CD24, CD49f, CD45, and CD31, in addition to Lrp5 and PI for live cell discrimination. PI positive, CD45+, and CD31+ cells were gated out prior to analysis of CD49f/CD24 profiles.

To determine Lrp5 and Lrp6 staining specificity by flow cytometry, 293 cells were transfected with plasmids encoding *Lrp5* or *Lrp6*. Following transfection, the cells were cultured for three days and removed from culture by incubating the cells approximately five minutes in PBS at 37 °C. The cells were then fixed and permeabilized using a Cytofix/Cytoperm kit (BD Biosciences), according to manufacturer instructions. The cells were then stained for Lrp5 or Lrp6 by incubating with each respective antibody for 30 minutes on ice. The cells were then washed and incubated with goat-anti-mouse-Alexa 488 (Molecular Probes) for 30 minutes on ice. The cells were washed and analyzed using a FACSCalibur flow cytometer (Becton Dickson).

***Immunofluorescence-*** 293 cells were transfected with plasmids encoding *Lrp5* or *Lrp6* and cultured for approximately 3 days. The cells were then fixed in 4% paraformaldehyde for 30 minutes at room temperature. The cells were washed and permeabilized in 0.01% triton X for 15 minutes and blocked in 10% normal goat serum for 30 minutes. The cells were stained for Lrp5 or Lrp6 by incubating overnight at 4 °C with primary antibodies. The cells were the stained with a goat anti-mouse-Alexa 546 (Molecular Probes) for two hours at room temperature prior to counter-staining with the nuclear dye, ToPro3 (Molecular Probes) for 30 minutes at room temperature. Images were taken at 200x, using a confocal microscope (BioRad).

***Western Blot Analysis-*** 293 cells were transfected with plasmids encoding *Lrp5*, *Lrp6*, or *GFP* and cultured for three days. The cells were removed from culture by scraping into culture medium and were centrifuged at 160 x g for 5 minutes. The pellets were resuspended in Streuli lysis buffer (50 mM Tris (pH 7.4), 150 mM NaCl, 2 mM EDTA, 1.5 mM Na_3_VO_4_, and 1% Triton X) and incubated for 30 minutes on ice. The lysates were centrifuged at 12,000 x g for 30 minutes. The supernatants were collected and protein concentrations estimated using Bradford reagent. Approximately 20 μg protein were loaded per lane into 4-20% SDS gels (Pierce, Rockford, IL), electrophoresed, and transferred onto PDVF membranes (Millipore, Billerica, MA). The resulting blots were blocked for 1 hour at room temperature in 10% nonfat milk and probed for Lrp5 or Lrp6 by incubating with primary antibodies 2 hours at room temperature. The blots were washed and incubated with goat-anti-mouse-HRP secondary antibody for 1 hour at room temperature. The blots were incubated for one minute with ECL substrate (Pierce) and exposed to film for varying lengths of time. Actin was used as a loading control.
